# Osteoclastic Giant Cell Rich Squamous Cell Carcinoma of the Uterine Cervix: A Case Report and Review of the Literature

**DOI:** 10.1155/2014/415328

**Published:** 2014-12-22

**Authors:** Lucía Alemán-Meza, Gabriela Sofía Gómez-Macías, Oralia Barboza-Quintana, Raquel Garza-Guajardo, Abelardo Loya-Solis

**Affiliations:** Pathology Department, University Hospital “Dr. Jose E. Gonzalez” and the Autonomous University of Nuevo Leon Medical School, Francisco I. Madero and Gonzalitos, 64460 Monterrey, NL, Mexico

## Abstract

Cervical carcinoma is the most common malignancy of the female genital tract and represents the second most common malignancy in women worldwide. Histologically 85 to 90% of cervical cancers are squamous cell carcinoma. Osteoclastic giant cell rich squamous cell carcinoma is an unusual histological variant of which only 4 cases have been reported. We present the case of a 49-year-old woman with a 6-month history of irregular vaginal bleeding. Examination revealed a 2.7 cm polypoid mass in the anterior lip of the uterine cervix. The patient underwent hysterectomy with bilateral salpingo-oophorectomy. Microscopically the tumor was composed of infiltrative nests of poorly differentiated nonkeratinizing squamous cell carcinoma. Interspersed in between these tumor cells were numerous osteoclastic giant cells with abundant eosinophilic cytoplasm devoid of nuclear atypia, hyperchromatism, or mitotic activity. Immunohistochemistry was performed; CK and P63 were strongly positive in the squamous component and negative in the osteoclastic giant cells, while CD68 and Vimentin were strongly positive in the giant cell population and negative in the squamous component. The patient received chemo- and radiotherapy for recurrent disease identified 3 months later on a follow-up CT scan; 7 months after the surgical procedure the patient is clinically and radiologically disease-free.

## 1. Introduction

Cervical carcinoma is the most common malignancy of the female genital tract and represents the second most common malignancy in women worldwide [1]. Histologically 85 to 90% of cervical cancers are squamous cell carcinoma [2]. Osteoclastic giant cells have been reported mainly in pancreatic [3] and breast carcinomas [4] and more rarely in liver [5], kidney [6], and urinary bladder [7] malignancies. Osteoclastic giant cell rich squamous cell carcinoma is an histologic variant not yet recognized by the World Health Organization (WHO) [8]; however it has been previously reported in 4 patients [9–11]; therefore its prognostic significance is currently unknown.

## 2. Case Report

**Figure 1:**
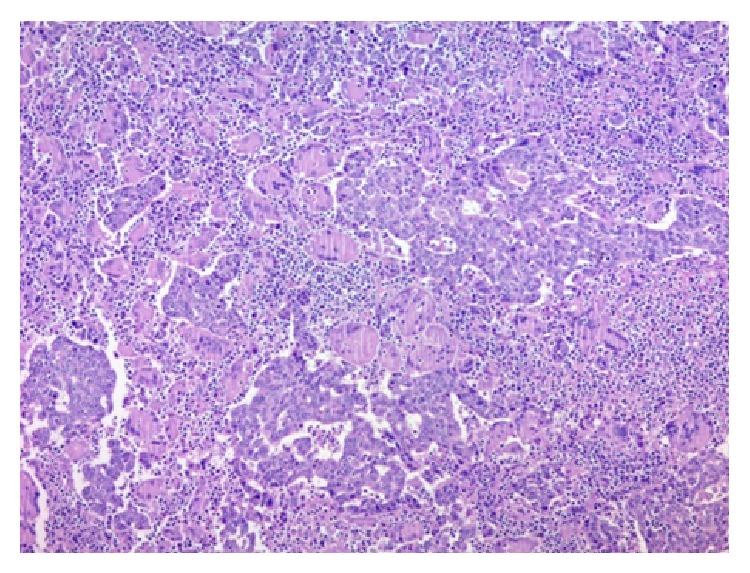
Nests of poorly differentiated squamous cells with interspersed osteoclastic giant cells. H&E stain, ×50.

**Figure 2:**
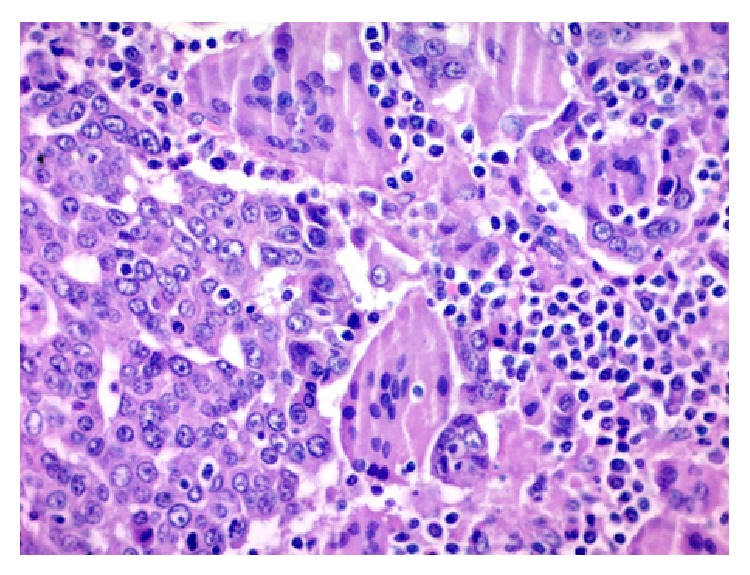
Osteoclastic giant cells with bland nuclei and lack of atypia. There is also an accompanying lymphocytic infiltrate. H&E stain, ×400.

**Figure 3:**
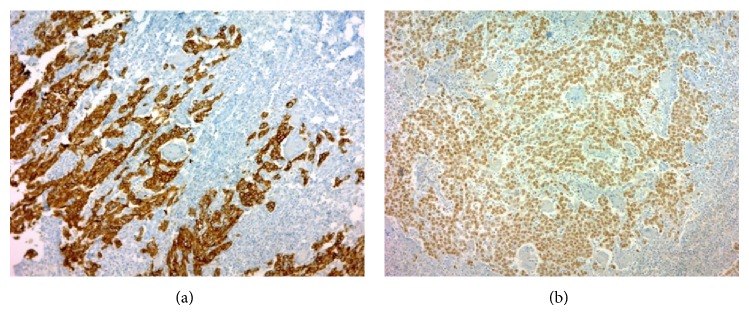
(a) Reactivity to cytokeratin in the squamous component, lack of reactivity in osteoclastic giant cells. Immunohistochemical stain with anti-CK antibody, ×50. (b) Reactivity to P63 in the squamous component, lack of reactivity in osteoclastic giant cells. Immunohistochemical stain with anti-P63 antibody, ×50.

**Figure 4:**
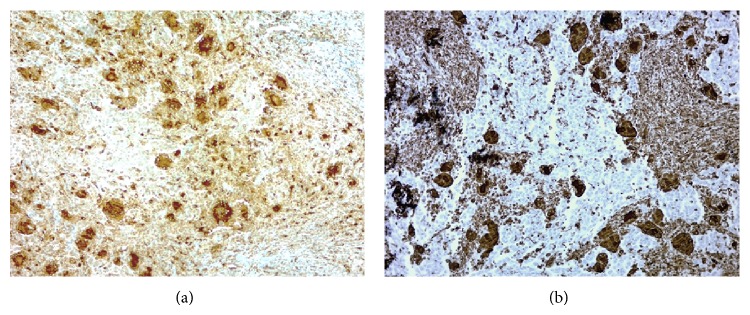
(a) Reactivity to CD68 in the giant cell population and lack of reactivity in the squamous component. Immunohistochemical stain with anti-CD68 antibody, ×50. (b) Reactivity to Vimentin in the giant cell population and lack of reactivity in the squamous component. Immunohistochemical stain with anti-Vimentin antibody, ×50.

**Figure 5:**
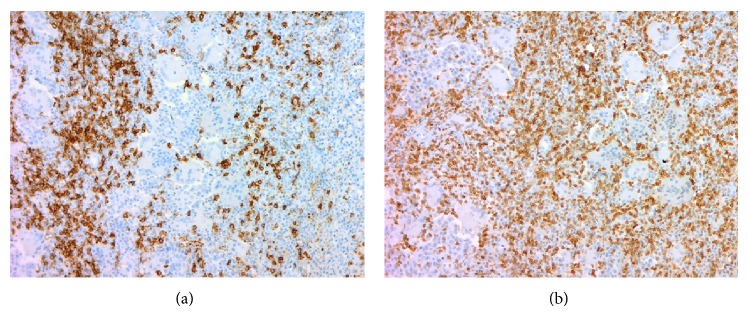
Reactivity to CD20 (a) and CD3 (b) showed a reactive pattern of the lymphocytic infiltrate. Immunohistochemical stain with anti-CD20 (a) and anti-CD3 (b) antibodies, ×100.

A 49-year-old woman consulted for assessment and management of a 6-month history of irregular vaginal bleeding associated with weight loss. Upon examination a 2.7 cm polypoid mass was identified in the anterior lip of the uterine cervix, a Papanicolaou smear was performed, and a squamous cell carcinoma was diagnosed. A CT scan disregarded extension of the mass to uterine corpus, abnormal lymph nodes, or distant metastasis. The patient was classified as FIGO Stage I (according to the International Federation of Gynecology and Obstetrics staging system) and underwent hysterectomy with bilateral salpingo-oophorectomy. Gross examination of the uterus showed a 2.7 × 2.5 tumor confined to the cervix. The adnexa were unremarkable. Microscopically the tumor was composed of infiltrative nests of poorly differentiated nonkeratinizing squamous cell carcinoma (Figure 1). Interspersed in between these tumor cells were numerous osteoclastic giant cells with abundant eosinophilic cytoplasm devoid of nuclear atypia, hyperchromatism, or mitotic activity; also there was an accompanying lymphocytic infiltrate (Figure 2). Margins were not involved and lymphovascular invasion was identified. Immunohistochemistry was performed using P63, cytokeratin (pankeratin-CK), Vimentin, CD68, CD20, and CD3. CK and P63 were strongly positive in the squamous component and negative in the osteoclastic giant cells (Figure 3), while Vimentin and CD-68 were strongly positive in the giant cell population and negative in the squamous component (Figure 4). Expression of CD20 and CD3 showed a reactive pattern of the lymphocytic infiltrate (Figure 5). Three months after the surgical procedure follow-up examination and CT scan showed recurrent disease in the region of the vaginal vault; thus the patient underwent chemotherapy (5 cycles) and radiotherapy (5000 cGy in 25 fractions). Two months after chemo- and radiotherapy (five months after the surgical procedure) a follow-up CT scan showed complete regression of the recurrent disease and disregarded local or distant metastases. Currently, seven months after the surgical procedure, the patient continues to be clinically disease-free.

## 3. Discussion

The conditions of the uterine cervix in which a giant cell component can be found, besides squamous cell carcinoma, include malignant mixed Müllerian tumors, high-grade sarcomas, choriocarcinoma, and tuberculosis [10]. The morphology of the giant cells in all of these conditions is very different and makes it possible to easily distinguish them. Malignant mixed Müllerian tumor and high grade sarcoma giant cells are characterized by nuclear pleomorphism. Tuberculosis giant cells, also known as Langhans giant cells, contain nuclei arranged in a horseshoe-shaped pattern in the cell periphery. Primary nongestational choriocarcinoma of the uterine cervix, although rare, has been reported [12] and consists of a mixture of multinucleated syncytiotrophoblastic giant cells with smudged, atypical nuclei and densely eosinophilic cytoplasm and mononucleated trophoblastic cells with clear cytoplasm. The osteoclastic giant cells found in squamous cell carcinoma have uniform nuclei and do not show any mitotic activity [10, 11].

The nature and prognostic implications of the osteoclastic giant cells in malignancies are still not clear. Their lack of atypia, hyperchromatism, and mitotic activity and their reactivity to Vimentin and CD68 with lack of reactivity to epithelial markers are indicative of a reactive histiocytic nature. Concerning prognostic implications, in pancreatic carcinoma their presence was previously associated with a better prognosis than ductal carcinoma [13], but more recent reports have suggested otherwise [14]. In the particular case of their presence in squamous cell carcinoma of the uterine cervix, no consensus yet can be achieved due to limited experience. Yu et al. believe their presence to be an indicator of poor prognosis [11], while Singh et al. believe them to be just a reactive component with no individual prognostic significance [10].

To the best of our knowledge, only 4 cases of osteoclastic giant cell rich squamous cell carcinoma of the uterine cervix have been reported [9–11], and our case is the first report from a Western country. All of the previous reports were from females over 60 years of age (mean age = 67.5), with a median tumor size of 5 cm and an IB2 FIGO stage. Three patients died within 14 months after treatment, and the remainder survived with complete tumor regression at the 6-month follow-up. Our patient is at least a decade younger than the mean age of the patients from previous reports and presented with a smaller tumor size as well as an earlier tumor stage (IB1). Her clinical outcome has also been, so far, better than that of the patients from previous reports.

In summary, osteoclastic giant cell rich squamous cell carcinoma of the uterine cervix is an unusual squamous cell carcinoma histologic variant not yet recognized by the WHO, characterized by the presence of an osteoclastic giant cell component of reactive histocytic nature. Its prognostic significance has not been fully established due to lack of experience. Further studies with larger number of patients and long term monitoring are required to establish this.
